# A New Method for the Fast Analysis of Trihalomethanes in Tap and Recycled Waters Using Headspace Gas Chromatography with Micro-Electron Capture Detection

**DOI:** 10.3390/ijerph14050527

**Published:** 2017-05-13

**Authors:** Lydon D. Alexandrou, Barry J. Meehan, Paul D. Morrison, Oliver A. H. Jones

**Affiliations:** 1 Australian Centre for Research on Separation Science (ACROSS), School of Science, RMIT University, GPO Box 2476, Melbourne, VIC 3001, Australia; s3284226@student.rmit.edu.au (L.D.A.); paul.morrison@rmit.edu.au (P.D.M.); 2 School of Science, RMIT University, GPO Box 2476, Melbourne, VIC 3001, Australia; barry.meehan@rmit.edu.au

**Keywords:** trihalomethanes, disinfection by-products, headspace, gas chromatography, separation science

## Abstract

Chemical disinfection of water supplies brings significant public health benefits by reducing microbial contamination. The process can however, result in the formation of toxic compounds through interactions between disinfectants and organic material in the source water. These new compounds are termed disinfection by-products (DBPs). The most common are the trihalomethanes (THMs) such as trichloromethane (chloroform), dichlorobromomethane, chlorodibromomethane and tribromomethane (bromoform); these are commonly reported as a single value for total trihalomethanes (TTHMs). Analysis of DBPs is commonly performed via time- and solvent-intensive sample preparation techniques such as liquid–liquid and solid phase extraction. In this study, a method using headspace gas chromatography with micro-electron capture detection was developed and applied for the analysis of THMs in drinking and recycled waters from across Melbourne (Victoria, Australia). The method allowed almost complete removal of the sample preparation step whilst maintaining trace level detection limits (>1 ppb). All drinking water samples had TTHM concentrations below the Australian regulatory limit of 250 µg/L but some were above the U.S. EPA limit of 60 µg/L. The highest TTHM concentration was 67.2 µg/L and lowest 22.9 µg/L. For recycled water, samples taken directly from treatment plants held significantly higher concentrations (153.2 µg/L TTHM) compared to samples from final use locations (4.9–9.3 µg/L).

## 1. Introduction

Disinfection by-products (DBPs) are formed through interactions between disinfectants and organic matter during the chemical treatment of water [1]. Currently, there are >600 known disinfection by-products [2], with this number increasing each year. A common class of DBPs are the trihalomethanes (THMs), which are readily formed through the treatment of waters containing organic matter [3] and via the breakdown of other DBPs [4,5]. This group is generally limited to the four most commonly observed species: trichloromethane (TCM), bromodichloromethane (BDCM), dibromochloromethane (DBCM) and tribromomethane (TBM) [6]. These four compounds are the basis for current THM regulatory limits, most commonly reported as the total trihalomethane (TTHM) concentration [7], which is the sum of the concentration of the above mentioned compounds [8]. Recently however, there has been a surge in interest in other THMs, such as iodinated and brominated compounds, increasing the number of potential THMs observed [9,10].

Most DBPs are highly toxic and thus strictly regulated in drinking water, although regulatory limits differ greatly between countries. Australia has a limit of 250 µg/L TTHMs; other countries are stricter and range from 60 µg/L (U.S.) to 100 (EU) µg/L [11]. The analysis of THMs is commonplace for drinking water to ensure compliance with these regulatory limits, and there are a number of standardised methodologies used for their analysis [6]. The more common analytical methods include direct aqueous injections, extraction techniques such as liquid–liquid extraction [12,13] and solid-phase extraction [14], solid-phase micro-extractions [15], dynamic headspace techniques (purge and trap) [16] and other headspace techniques [4].

In the case of THM analysis, the above methods are all widely used, dependent on access to equipment, though all have drawbacks. Liquid-liquid extractions generally have a higher contamination risk, due to direct interaction with multiple solvents. Solid-phase extractions have low recoveries and are commonly slower, thereby increasing potential sample loss, though this can be mitigated with smaller scale extractions. Solid-phase micro-extractions though relatively cheap and effective, are mostly perceived as semi-quantitative but are commonly employed due to their ease of use. Dynamic headspace techniques are the most widely used method, but are also more time consuming than other methods, whilst also highly dependent on specific equipment set-up. Static headspace techniques have a wide application due to their potential higher selectivity (under optimized conditions) but are less commonly used compared to dynamic headspace methods which are more flexible in application.

In the past, static headspace techniques have been frequently shown to yield high sensitivities for DBPs due to the latter’s inherent volatility, with the majority of these methods using solid-phase micro-extraction (SPME) [6]. An alternate to the SPME is the use of a direct headspace technique. This removed the need for an optimised SPME fibre, relying instead on the volatility of the target analytes [17,18]. This method does increase total analysis time as it requires an agitation/heating period as well as chromatographic separation but this step can be fully automated and the method as a whole requires minimal sample preparation. This means it can provide a quick and simple, yet accurate, analysis from a raw sample.

The aim of the present study was to develop a direct headspace technique for the rapid analysis of THMs, without the need for prior sample preparation and then apply this method to drinking water and recycled waters in and around Melbourne (VIC, Australia).

## 2. Materials and Methods

### 2.1. Chromatographic Separation

Sample analysis was performed using an Agilent 6890 series gas chromatograph (GC) with micro-electron capture detection (µECD). Chromatographic separations were completed using a SGE BP264 column (30 m × 220 µm film thickness) obtained from SGE Analytical science (Ringwood, VIC, Australia) and a split ratio of 10:1. The instrumental operating conditions are shown in Table 1.

### 2.2. Reagents

#### 2.2.1. Reagent Water

Standards and blanks were prepared from ultrapure water at 18 mΩ using an Ultrapure water system (Millipore, North Ryde, NSW, Australia). The water was placed in an ultrasonic bath and sonicated for 1 h before use to remove any residual volatile components that might have interfered with analysis.

#### 2.2.2. Standard Solutions

Stock solutions were prepared from standards solutions (all in HPLC grade solvents (≥98 % Purity) of each of the four target THMs and the internal standard. Individual standard solutions of bromodichloromethane (BDCM), dibromochloromethane (DBCM), tribromomethane (TBM) and 1,2-dibromopropane (1,2-DBP) were purchased from Sigma-Aldrich (Castle Hill, NSW, Australia), while trichloromethane (TCM) and methanol (MeOH) were purchased from Merck Millipore (Kilsyth, VIC, Australia).

Primary stock solutions were prepared individually by weight for each of the analytes, using the primary stock solutions to prepare a standard mixture of the four THMs at 100 mg/L in MeOH, alongside an individual standard of the internal standard (1,2-DBP) at the same concentration. Intermediate stock solutions at 2, 1, 0.1, 0.01 mg/L in MeOH were prepared by the dilution of the standard mixture with MeOH (internal standard intermediate prepared at 1 mg/L in MeOH).

Calibration standards were prepared from the stock solutions directly into 20 mL vials in a range of 0.1 to 100 µg/L. The total volume of each sample was 10 mL leaving a headspace volume of 10 mL. All samples were spiked to a level of 10 µg/L of the internal standard (diluted from 1 mg/L intermediate stock solution). Parallel to creation of the calibration standards, spiked standards were prepared using drinking water and environmental waters and analyzed to test for any potential matrix effects. All spiked standards were sonicated before addition to remove residual THMs. Due to the similarity between ultrapure water and drinking water, there were no observed matrix effects other than residual THM presence in the potable samples.

Individual standards of each THM were also tested to identify their retention times. To ensure the accuracy of standard preparation, calibration standards were run on multiple occasions during method development and application.

### 2.3. Water Sample Collection and Preparation

**Figure 1:**
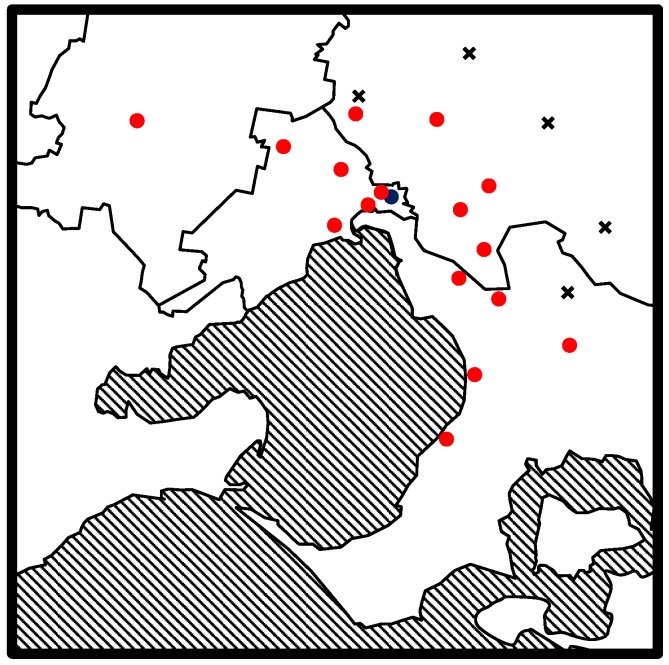
Drinking water sample locations (dots) and the water treatment plants (crosses) that distribute drinking water to suburban Melbourne (Victoria, Australia). Bordered areas are the sectors serviced by the main water utilities in Melbourne.

In order to test this method on real environmental samples, samples from a variety of water sources that had undergone chemical treatment, were tested. Sampling was completed on a wide range of tap water samples from across Melbourne, Australia. The sampling locations can be seen in Figure 1.

Drinking samples were collected from residential taps directly into headspace sample vials. At each site, six water samples were collected. Three samples were collected as soon as the tap was turned on after having been left closed overnight and three samples were collected after the tap had been running for 10 min. At each sampling site, a field blank was also employed. This consisted of a sampling vial prefilled with sonicated Milli Q water containing no DBPs. This vial was left open throughout the period of water collection to test for background DBPs at each sampling site (NB: no background DBP contamination was found at any site).

Recycled water sources were also sampled alongside drinking water samples as a comparison. Recycled waters were collected from two inner city fountains in Melbourne. Class C and Class A recycled waters were also sampled directly from a water treatment plant in Eastern Melbourne. Class C and Class A water samples were collected directly from taps at the treatment works directly after the relevant treatment stage. Class C recycled water is the product of physical and biological treatment while Class A water is Class C water that has undergone chemical treatment, in this case chlorination. The recycled water samples were prepared in headspace vials in the same manner as the drinking water samples. All were analyzed within 24 h of collection.

### 2.4. Procedure for Headspace Extraction on Calibration Standards and Samples

Headspace extractions were completed using 20 mL headspace vials with magnetic screw top vial lids purchased from Agilent technologies (Mulgrave, VIC, Australia). Prior to use, each vial was baked at 150 °C for 60 min prior. Before injection, samples were heated at a temperature of 30 °C for 30 min and agitated at 500 rpm for 30 min. After the agitation/heating period, the vial headspace was sampled, using a 2.5 mL syringe (injection volume of 500 µL). Between aspiration and injection of the sample into the GC, the syringe was kept at a temperature of 35 °C. The syringe was flushed with N_2_ gas for 10 s between each sample. The extraction method was fully automated using vials with magnetic caps.

Prior to real-world sampling, standard mixes of other commonly occurring DBPs and halogenated volatiles were analyzed to identify potential interference peaks in any given chromatogram due to the high sensitivity and specificity (for halogenated compounds) of the µECD. The separation method was then optimized to account for other potential components.

Quantification of the four analytes was performed using peak area ratios of the analyte relative to the internal standard based on a multi-level calibration of 0.1 to 100 µg/L (relative peak area as a function of concentration). All standards and samples were analysed in triplicate.

## 3. Results

### 3.1. Linearity and Analyte Calibration

The linear range of the method was evaluated by plotting calibration curves of the relative peak area (analyte relative to the internal standard) for each analyte versus the concentration. Standard calibrations were plotted for concentrations ranging from 0.1 to 100 µg/L. The curves obtained showed linearity for each analyte across the calibration range. Concentrations above 250 µg/L were not analysed as any concentration above this level would exceed the current Australian regulatory limit for THMs (250 µg/L TTHMs).

### 3.2. Limits of Detection and Analysis Robustness

The sensitivity of the headspace method was considered in terms of limit of detection and limit of quantification (LOD and LOQ respectively). These calculations were conducted using nine replicate samples at an estimated method limit, as per the U.S. EPA’s method detection limit [19]. The results can be seen in Table 2. The repeatability for the nine replicate samples was also scrutinized, a %RSD for the target analytes of between 2.4 to 4.3% at the tested standard concentrations of 1 µg/L was observed, indicating that the method was performed properly.

**Figure 2:**
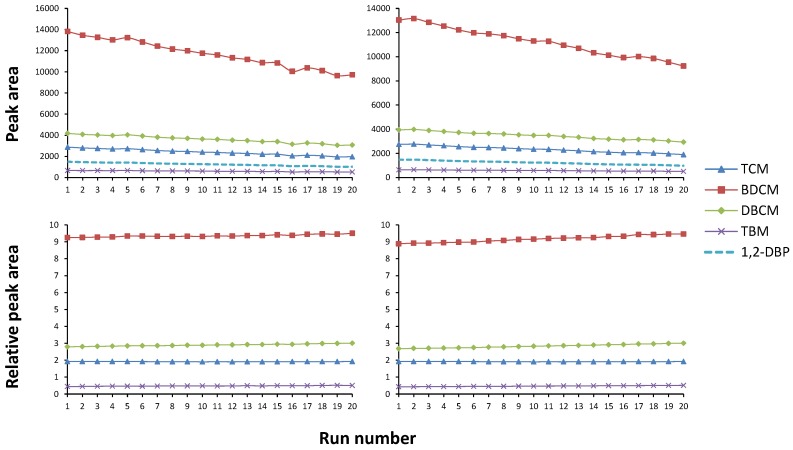
Consecutive runs of individual standards (each analyte 10 µg/L). Each sample was run 20 times in a row, with no interval between sample agitations (**left column**) and with 30 min intervals between agitations (**right column**). The upper plots show the peak area of each visible peak (four trihalomethanes (THMs) and internal standard) while the lower plots show the areas of the THMs when normalised to the internal standard.

Since THMs are highly volatile, the method was also analysed to test for potential sample loss due to the vial septa being pierced multiple times during analysis. For this test, two sets of standard runs were initiated in which a single sample was analysed 20 times in succession. The first set of runs was performed as the method would run (30 min agitation/heating). Then, the second set of runs allowed an extra 30 min between each agitation period. The observed analyte response was normalized to the internal standard. Graphs of the results of these tests can be seen in Figure 2.

### 3.3. Effect of Salt Addition

**Figure 3:**
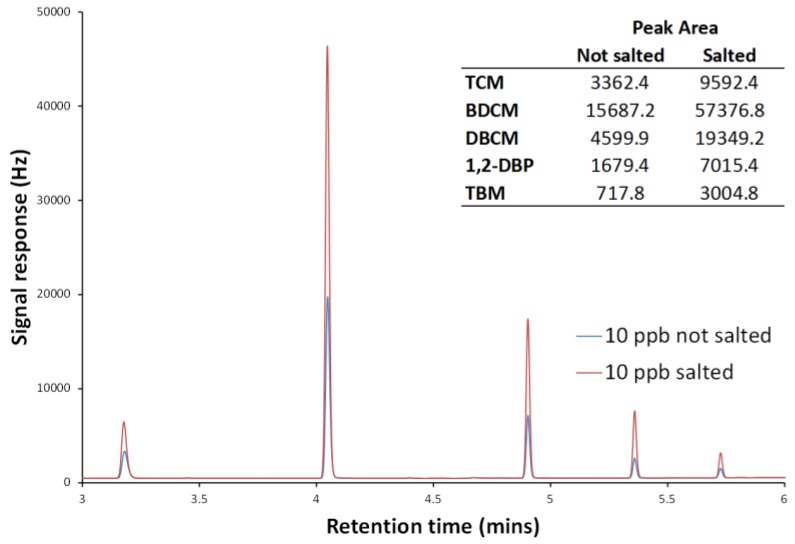
Chromatograms of spiked samples containing TCM (3.17 min), BDCM (4.04 min). DBCM (4.90 min), 1,2-DBP as internal standard (5.38 min) and TBM (5.75 min) with one sample salted and one not salted. Inset table indicates the peak areas of the target analytes in both samples.

Literature headspace methods, both dynamic and static, generally involve the addition of a salt before agitation/heating to increase volatilisation of the target analytes [20]. To test the effect of salt addition, spiked standards were analysed after the addition and dissolution of salt (sodium sulphate) at varying masses, (dissolved masses of up to 2 g were tested). Using the maximum salt addition for a 10 µg/L sample, signal responses were increased by approximately a factor of 3–4. A comparison of spiked standards, with and without added salt, can be seen in Figure 3.

As the aim of the developed method was to remove sample preparation steps as far as possible, a salt addition was not used for the final method. Though the addition of salt greatly increases the analyte responses and thus the detection limits, there was also an increase in the background noise. In addition, the detection limit using non-salted samples was well below reported environmental concentrations so the extra sensitivity provided by salt addition was not needed.

### 3.4. Occurrence Studies

The analysed samples covered 17 metropolitan locations, as well as four recycled water sources. The metropolitan sources were residential drinking water taps, and were grouped according the location’s water supplier. This formed four groups (three groups of five locations; one group of two locations). In all samples, the signal responses for TCM, BDCM and DBCM were above both the LOQ and LOD.

## 4. Discussion

The method developed in this work was tested on samples collected from a variety of locations in Melbourne; the majority being drinking water, with some recycled water samples also tested for comparison. The samples were untouched from collection to analysis (no filtration or sample pre-concentration). Each sample was prepared and analyzed from the raw state in approximately 40 min. Taken together, the data show that the method used is quicker than existing methods and feasible for environmental applications.

In all samples, the signal responses for TCM, BDCM and DBCM were above both the LOQ and LODs. In the case of TBM, the compound was rarely observed. No samples were found to have concentrations greater than the Australian drinking water guidelines of 250 µg/L TTHM. For the tested areas (See Table 3), Group 1 had a high TTHM of 67 µg/L and low of 24 µg/L; Group 2 had a high of 45 µg/L and low of 26 µg/L; Group 3 had a high of 56 µg/L and a low of 35 µg/L.

Samples of Class A and Class C recycled water were collected directly from the treatment plants and therefore show the THM concentrations present at the completion of the appropriate stage of treatment. In the case of Class C water, the observed concentrations were low as expected (Class C water having not undergone any chemical treatment). Class A water concentrations were very high. Although still below the Australian guideline limits of 250 µg/L, they did in some cases exceed limits set elsewhere. Recycled waters sampled from inner city fountains had concentrations of approximately 5 µg/L TTHMs, indicating substantial loss of THMs during transit in the water distributing system and/or via volatilization due to sampling locations being in the open air.

Concentrations of THMs in drinking water samples from areas served by the same water utility (and which therefore would have been produced at the same plant) were similar, with minor differences arising due to distance of the sampling point from a given plant. In general, the further a sampling point was from the source plant, the higher the residence time and the greater the loss of analyte. However, it should be noted that water “age” can also be influenced by velocity in the pipes and water demands from the population. Increased residence time was also the most likely cause of a drastic difference in concentration of the recycled water samples compared to the drinking water samples since recycled water generally has a holding period before distribution to reduce levels of any residual pathogens that may be present.

An interesting trend was seen in the group 3 samples, where all five locations are from a single plant (the locations were all sampled within 3 days of each other). In this case, the trend for decreasing TCM levels with distance held true but there was an increase in the overall level of brominated THM species present. The increase in brominated species is odd as while the treatment process will add residual disinfectant to maintain water quality through transit, it never involves the introduction of free bromine. This result may be due to a bromine source (perhaps infiltration by groundwater) somewhere along the pipeline but more detailed and long-term analysis would be needed to determine this.

It is worth considering that headspace methods can be hampered by background interference from other DBPs present in the sample since most DBPs are highly volatile and can be released from solution during the extraction. The major interference in the case of THMs is that of haloacetic acids and haloacetonitriles, both of which have been shown to break down into THMs [4], providing false readings of both groups. In the present study, the volatilisation of other DBPs was eliminated by using a lower temperature and reduced agitation time and salt concentration than typical headspace methods [20,21]. This also allowed a much shorter analysis time of 10 min compared to the 20–30 min runtimes that are common for the analysis of multiple DBPs.

## 5. Conclusions

A headspace gas chromatography (HS-GC) was developed and applied to the analysis of trihalomethanes (THMs) in drinking and recycled waters. The aims of this work were to (i) develop a new direct headspace technique for the rapid analysis of THMs that removed or reduced the need for prior time-consuming sample preparation and (ii) to apply the developed technique to raw drinking water and recycled water samples. The method was successfully developed to reduce or remove typical sample preparation steps, such as salt addition, pre-concentration and filtration, to analyse samples directly from the source with minimal external influences. A sample volume of 10 mL (1:1 sample: headspace ratio) with an extraction time of 30 min at 30 °C were the observed optimal conditions for the analysis of THMs. The method achieved limits of detection below 0.5 µg/L for the four analysed compounds (trichloromethane, dichlorobromomethane, bromodichloromethane and tribromomethane) in a variety of sample matrices. The total trihalomethane concentrations in the tested drinking water samples did not exceed the Australian regulatory limits of 250 µg/L, but were in some cases, just above the U.S. EPA limit of 60 µg/L. Through the use of this headspace method, sample preparation time was reduced to less than 1 min per sample, achieving a high throughput method that has great potential for the rapid analysis of THMs in drinking and recycled waters.

## Figures and Tables

**Table 1:** Gas chromatograph operating conditions.

Parameter	Conditions
Injection volume	500 µL
Carrier Gas	H2, 1.6 mL/min, constant flow
Make-up gas	Nitrogen, 60 mL/min
Split ratio	10:1
Injector temp	220 °C
Detector temp	230 °C
Oven program	35 °C for 1 min, then 25 °C/min to 230 °C held for 1.20 min(total runtime 10 min)

**Table 2:** Limits of detection (LOD) and limits of quantification (LOQ) for the target analytes.

Analyte	LOD (ppb)	LOQ (ppb)
Trichloromethane (TCM)	0.14	0.47
Bromodichloromethane (BDCM)	0.09	0.32
Dibromochloromethane (DBCM)	0.10	0.35
Tribromomethane (TBM)	0.14	0.47

**Table 3:** Summary of THM occurrence data in analysed drinking and recycled waters.

Group	Sample Location	Analyte Concentrations (µg/L) (%RSD)				
		TCM	BDCM	DBCM	TBM	TTHMs
**Drinking Water**						
1	1	19.6 (5.1)	4.0 (4.3)	1.6 (2.3)	ND	25.2
	2	20.7 (1.6)	8.6 (1.5)	6.7 (1.1)	0.7 (57.6)	36.7
	3	47.3 (1.9)	4.7 (1.1)	0.9 (0.4)	ND	52.9
	4	56.4 (3.6)	4.9 (2.8)	0.9 (0.7)	ND	62.2
	5	56.4 (3.6)	4.9 (2.8)	0.9 (0.7)	ND	62.2
2	6	35.5 (3.1)	6.1 (2.7)	3.1 (2.4)	ND	44.8
	7	21.9 (1.6)	4.4 (1.1)	1.7 (0.8)	ND	28.0
	8	26.6 (5.6)	8.9 (5.2)	6.1 (2.8)	ND	41.7
	9	21.6 (5.4)	9.3 (4.8)	7.1 (3.0)	1.0 (2.2)	39.0
	10	17.9 (1.8)	10.5 (1.6)	8.3 (1.6)	1.1 (1.2)	38.0
3	11	29.4 (1.8)	7.2 (1.5)	2.2 (1.6)	ND	38.9
	12	57.6 (3.0)	5.3 (1.8)	1.0 (0.5)	ND	63.9
	13	27.6 (0.9)	7.0 (0.3)	2.3 (0.2)	ND	36.9
	14	27.3 (0.7)	7.1 (1.1)	2.3 (0.8)	ND	36.8
	15	35.0 (2.5)	15.1 (1.3)	10.0 (1.0)	1.2 (1.1)	61.2
4	16	39.9 (1.3)	6.1 (0.3)	2.4 (1.9)	ND	48.4
	17 *	28.2 (4.6)	8.3 (3.3)	5.3 (8.8)	ND	41.8
		31.1 (1.5)	7.6 (1.2)	4.3 (2.6)	ND	43.0
		29.2 (3.7)	7.6 (1.7)	4.5 (5.7)	ND	41.3
**Recycled Water**						
	Fountain 1	1.5 (2.9)	1.1 (0.9)	1.7 (1.5)	5.0 (1.7)	9.3
	Fountain 2	2.2 (0.7)	1.4 (0.5)	1.3 (0.6)	ND	4.9
	Class C water	2.8 (0.9)	0.7 (0.4)	ND	1.0 (4.4)	4.5
	Class A water	117.6 (3.2)	27.1 (2.2)	7.2 (2.9)	1.4 (6.6)	153.2

* Tap water was sampled and analysed on multiple days, ND = Not detected.
